# Comment on Chiu et al. Health Promotion and Disease Prevention Interventions for the Elderly: A Scoping Review from 2015–2019. *Int. J. Environ. Res. Public Health* 2020, *17*, 5335

**DOI:** 10.3390/ijerph19095271

**Published:** 2022-04-26

**Authors:** Tim Neumann

**Affiliations:** BIH QUEST Center for Responsible Research, Berlin Institute of Health (BIH) at Charité, Universitätsmedizin Berlin, 10178 Berlin, Germany; tim.neumann@charite.de

Chiu et al. report in their paper “Health Promotion and Disease Prevention Interventions for the Elderly: A Scoping Review from 2015–2019” [1] that the amount of literature promoting health for the elderly has increased, particularly that addressing health promotion, screening, primary prevention, and social support. They also report that health promotion activities to prevent the elderly from falling and to improve home safety and mental health promotion do use eHealth technology. They also conclude that the use of eHealth technology it is still immature, “thus more rigorous research is needed in different areas, especially in older populations, various professions, women, and people with dementia”.

In my opinion, the conclusions as published are not justified in the current wording. It is not justified to speak about “the amount of literature” (Abstract) as long as the search was limited to the “secondary literature” such as systematic reviews and/or meta-analyses. This means that some of the relevant literature, especially new evidence derived from RCTs, experimental studies, cohort studies, etc., was not considered. Additionally, one original paper might have been considered in several reviews. This is a limitation. It is necessary to mention in the title that this is a “Scoping review of reviews”, and to mention or describe the limitations in the abstract, methods, result and discussion, including the limitation section. The words “study”, “review”, “meta-analysis”, “scoping review”, and “paper”, “literature, “articles”, etc., should be used precisely and be understandable, comprehensible and unambiguous. This current use might be misleading. The study methodology is also not designed for the “purpose of identifying and reviewing the effectiveness of different interventions addressing elderly health promotion and related areas”, as outlined in the first lines of the Methods chapter.

Experienced readers might recognize the misleading choice of words in this context. Relevant points are addressed below:

It would be helpful to use “scoping review” or “scoping review of reviews” instead of “study”, if it is designating a scoping review (Abstract lines 1, 3, 5; page 2: lines 4, 19, 20, 28, 34, 36, 37; page 3: line 42; page 7:, lines 2, 4, 5, 7, 28; page 8: lines 39, 48; page 9: lines 45, 48; page 10: line 19). 

However, the use of the word “study” is inconsistent: The word “study” is also used to designate a “review” (systematic review, meta-analysis), included in the scoping review (of reviews), which is confusing (Abstract: line 4; page 3: line 42; page 4: lines 10, 11, 17, 18, 23; page 5: line 10, page 6: lines 8, 9, page 7: lines 4, 11,12, 32, page 8: lines 7, 8, 20, 22, 24, 36, 43, 48, page 9: lines 3, 26, 27, 32, 35, 37, 45, 46, page 10: lines 3, 15, 18). This is also true for the use of the word “paper” (Figure 1) or “articles” (page 7: lines 7, 8, 9), which is used for the same meaning. 

If reviews/meta-analyses or secondary literature are meant, using the words “research” (page 2 lines 5, 15, 28, page 4, last line, page 7, line 10) and “literature” (page 2, line 19; page 7, 3rd last line, abstract line 6) is misleading. The included systematic reviews and meta-analyses are not “the research on health promotion” (Page 7, lines 9, 10; Abstract line 2).

Page 7, line 16: “Health promotion” is not a “research method” for “health promotion, screening primary prevention and social support”.

The paper could have some merits. Therefore, these clarifications are of interest as only a “rigorous peer-review process together with strict ethical policies and standards” can “ensure the addition of high-quality scientific studies to the field of scholarly publication”, as mentioned on the homepage of this journal [2,3].
